# Incorporation of Poly(TFEMA) in Perovskite Thin Films Using a Supercritical Fluid

**DOI:** 10.3390/molecules28145385

**Published:** 2023-07-13

**Authors:** Kasey Handy, Gary C. Tepper

**Affiliations:** Department of Mechanical and Nuclear Engineering, Virginia Commonwealth University, Richmond, VA 23284, USA; handyk2@vcu.edu

**Keywords:** poly(TFEMA), perovskite, supercritical CO_2_

## Abstract

A new process is reported for the incorporation of a fluoropolymer into a solid perovskite film. Poly(trifluoroethyl methacrylate) [CH_2_C(CH_3_)(CO_2_CH_2_CF_3_)]_n_ was delivered to methylammonium lead iodide (CH_3_NH_3_PbI_3_) perovskite films by crystallizing the film in supercritical carbon dioxide/ethanol containing the dissolved fluoropolymer. The surface was characterized before and after fluoropolymer exposure using scanning electron microscopy, Raman spectroscopy, and contact angle measurements. The results indicate that the fluoropolymer was incorporated into the perovskite film during the supercritical fluid crystallization process. The incorporation of a hydrophobic fluoropolymer into perovskite has the potential to improve resistance to environmental degradation.

## 1. Introduction

Perovskite-based solar cells offer the potential of a new renewable energy technology capable of efficiently converting sunlight into electricity with the benefit of cost-effective manufacturing from solution. Organic–inorganic metal halide perovskite solar cells have achieved photoconversion efficiencies exceeding 25% as of 26 January 2022 [1]. The high conversion efficiency is due to the excellent electronic and optical properties, including high absorption [2], small exciton binding energy [3], high carrier mobility [4], large carrier diffusion length [5], bandgap tenability [6], and high tolerance for defects [7,8]. The primary challenge limiting the commercialization and broad use of perovskite cells is performance degradation under exposure to environmental factors such as humidity, temperature, and radiation.

Organic–inorganic hybrid perovskites such as methylammonium lead iodide (CH_3_NH_3_PbI_3_) have an ABX_3_ crystal structure, where A is an organic cation such as methylammonium or formamidinium, B is an inorganic cation such as lead or tin, and X is a halide or oxide anion such as iodide, bromide, or chloride [9,10]. Materials with this structure exhibit unusually good photoelectric and transport properties, making them ideally suited for solar energy harvesting [11,12]. Material properties such as bandgap and density can be tuned through the appropriate selection of the ABX_3_ components in order to optimize processing and performance.

One of the most important advantages of CH_3_NH_3_PbI_3_ perovskite materials is the ability to deposit from solution. Common methods include one- or two-step solution deposition. The one-step solution deposition method involves spin coating a solution containing organic and inorganic perovskite precursor components on a substrate and then annealing to form perovskite. The two-step solution-based method is similar, except there are two spin coating steps, the first solution being an inorganic component and the second an organic component followed by annealing. Other, but less common, deposition methods include sequential vapor deposition, vapor-assisted solution processing, and vacuum deposition [13,14,15]. The main concerns during fabrication are film quality, uniformity, and reproducibility. From a manufacturing point of view, production at large scale is difficult using spin-coating. New technologies meant to be compatible with manufacturing include roll–roll processing, spray-coating, doctor-blade coating, soft-cover deposition, drop casting, ultrasonic coating, and electrospray [16,17].

Supercritical fluids have been widely used for the production of particles and nanostructured materials and for solvent extraction [18,19]. The supercritical state occurs when a fluid is brought above its critical point, creating a combination of gas and liquid-like properties (e.g., density, viscosity, diffusion coefficient). Supercritical CO_2_ (scCO_2_) is the most common since it is non-toxic, non-flammable, non-reactive, inexpensive, environmentally friendly, and energy efficient. It also has a moderate critical temperature and pressure of 31.1 °C and 73.8 bar, respectively. The thermophysical properties of scCO_2_ can be changed easily by adjusting the temperature and pressure, thus allowing scCO_2_ to be used as either the solvent, anti-solvent, solute, or reaction medium during processing [20].

Previously, our group has demonstrated the advantages of post-deposition annealing of perovskite films in scCO_2_ [21]. Perovskite films are typically deposited by dissolving precursor molecules in a solution and creating a film by a method such as spin coating. Post-deposition annealing is used to promote crystallization and to remove any residual solvent. We showed that scCO_2_ acts as an antisolvent during the solid-state film crystallization with the advantage that the fluid bathes the entire film for uniform annealing and does not leave any harmful solvent residue upon removal. Removal of scCO_2_ is achieved by isothermally decreasing the pressure of the supercritical fluid, allowing it to enter the gas phase without crossing a phase boundary [22]. Low-temperature annealing of perovskite in scCO_2_ accelerates the kinetics of solid-state crystallization and increases the average grain size of CH_3_NH_3_PbI_3_ films [21]. The combination of larger grains and enhanced crystalline quality improved the energy conversion efficiency of perovskite-based solar cells in comparison to those annealed at the same temperature without scCO_2_ [23].

While perovskite-based devices exhibit excellent photoconversion efficiency, the long-term performance can be rapidly degraded by exposure to environmental factors such as humidity. One of the most promising applications of a supercritical fluid during post-deposition treatment of perovskite films is the addition of chemical additives for defect passivation or surface modification for improved stability and resistance to environmental degradation. Supercritical fluids are widely used for the selective extraction of chemicals from materials and can also be used for infusion of chemical compounds into materials [19,24]. To deliver a polymer or other compound to a surface, the chemical can simply be added to the supercritical fluid as a solute and delivered to the surface during the post-deposition annealing process. Fluoropolymers are particularly suited for this application since they can be soluble in supercritical CO_2_ and are generally hydrophobic and moisture-resistant. The challenge is to apply the fluoropolymer such that it increases the moisture resistance of the perovskite film without interfering with light absorption or charge transport, which would decrease the photovoltaic performance of the solar cell. Therefore, the fluoropolymer should be integrated with the perovskite layer in locations such as grain boundaries to act as a defect-passivating agent rather than deposited as a separate layer.

Poly(TFEMA) was selected for this study because it is hydrophobic and can be dissolved in scCO_2_ [25,26]. A small amount of ethanol was added to scCO_2_ as a co-solvent to increase the poly(TFEMA) solubility for processing at lower temperatures and pressures. We previously studied the effect of scCO_2_/ethanol on the solid-state crystallization and resulting morphology of CH_3_NH_3_PbI_3_ perovskite films and demonstrated that the ethanol co-solvent, due to its preferential affinity for methylammonium, can be used to selectively modify the perovskite surface morphology [21].

**Figure 1:**
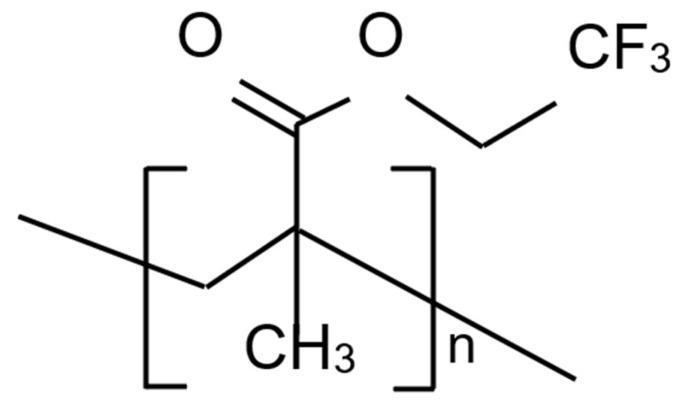
FTEMA monomer structure.

The Poly(TFEMA) molecular structure is shown in Figure 1. The three fluorine atoms are bonded to a carbon atom and extend outside of the main polymer backbone. This fluoropolymer is known to have excellent heat and chemical resistance, low refractive index, weatherability, non-cohesiveness, water and oil repellency, transparency, and electric insulating properties [26,27]. The polymer can be produced through free radical polymerization using bulk, solution, and emulsion polymerization methods with supercritical carbon dioxide as the polymerization medium [27]. A previous study reported on the deposition of Poly(TFEMA) particulate coatings using rapid expansion of supercritical solutions (RESS) [28]. In the RESS method, the solute is precipitated as the solution is depressurized across an orifice or nozzle and the substrate is not directly exposed to the supercritical solvent. Therefore, the deposition is performed in the gas phase resulting in polymer particles on the surface.

The objective of this experiment is to integrate poly(TFEMA) in CH_3_NH_3_PbI_3_ perovskite thin films during solid-state crystallization in a scCO_2_/ethanol solution. The solubility of poly(TFEMA) in scCO_2_/ethanol was determined by measuring the cloud point. Raman spectroscopy was used to characterize the perovskite film before and after fluoropolymer exposure and the surface morphology was imaged using scanning electron microscopy. Contact angle measurements were performed to characterize the surface wettability before and after fluoropolymer deposition. The results indicate that the fluoropolymer was incorporated into the perovskite film rather than appearing as a separate layer. The ability to incorporate a fluoropolymer or other chemical additive into perovskite has the potential to improve the long-term performance and resistance to environmental degradation.

## 2. Results and Discussion

**Figure 2:**
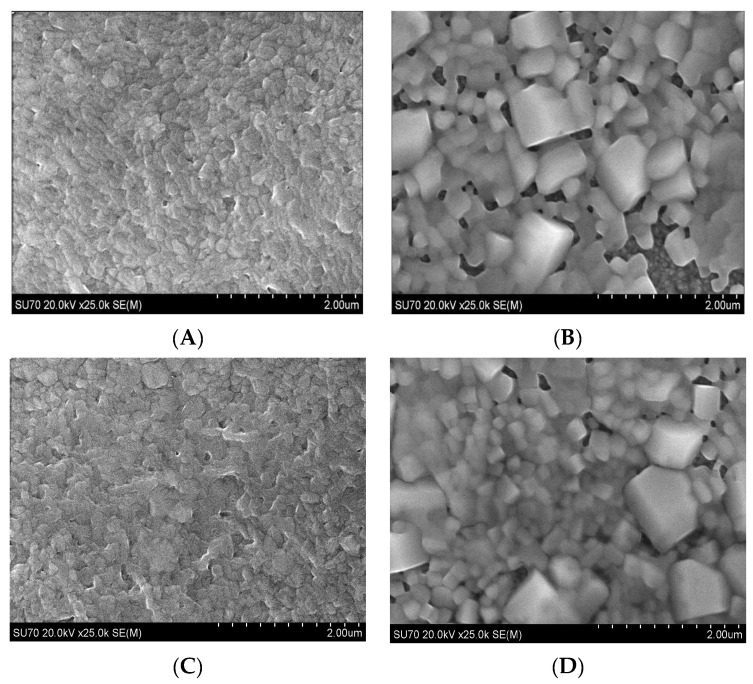
SEM images of (**A**,**C**) bare perovskite and (**B**,**D**) after poly(TFEMA) addition.

Figure 2A,C are SEM images of the bare perovskite film and Figure 2B,D are SEM images of the film after fluoropolymer exposure in the scCO_2_/ethanol solution according to the method of Section 3.4. The morphology of the bare perovskite is as expected and is consistent with the morphology our group previously reported for films annealed at 50 °C and consists of small, irregularly shaped grains [29]. The films in Figure 2B,D, after fluoropolymer exposure, are somewhat surprising. The films exhibit a morphology consisting of agglomerated cuboid particles ranging in size from about 200 nm to 1 micron. The morphology of Figure 2B,D is similar to the morphology we previously reported for perovskite films annealed in scCO_2_ and 2% ethanol without a fluoropolymer solute [21], but exhibits much greater particle agglomeration. There is no obvious presence of distinct fluoropolymer particles in either image. Solute particles would normally be expected to appear on the surface, since depressurization across the cloud point results in particle precipitation. The absence of visually distinct fluopolymer particles suggests that the poly(TFEMA) fluoropolymer was either not deposited onto the perovskite surface or was integrated into the perovskite film during the scCO_2_/ethanol anneal. The significant amount of particle agglomeration seen in the SEM image of Figure 2B,D was not observed in our previous study and suggests the possibility that the fluoropolymer might be present within the film, but not in the form of distinct particles.

**Figure 3:**
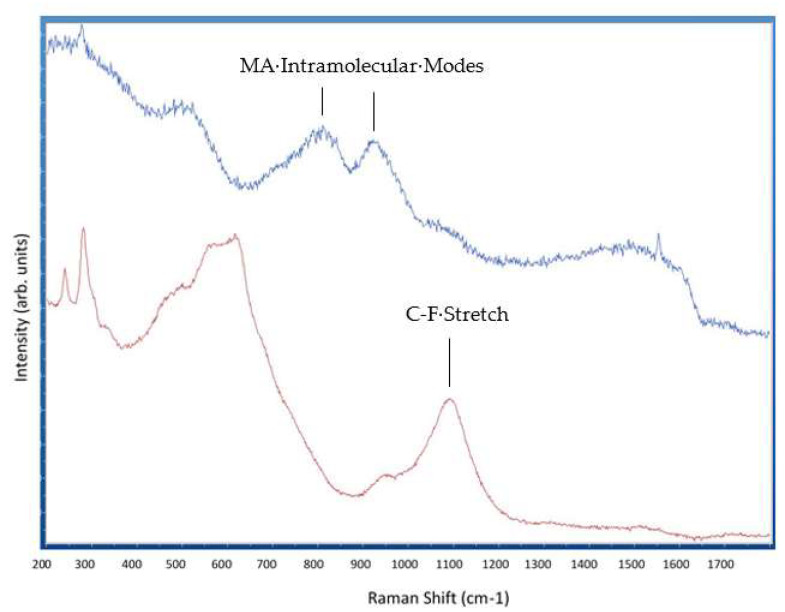
Raman spectra of bare perovskite film (**top**) and perovskite film after fluoropolymer deposition (**bottom**).

Raman spectroscopy was performed to characterize the surface chemical composition before and after the deposition of Poly(TFEMA). Figure 3 shows the Raman spectra measured at an excitation wavelength of 532 nm. The top line represents the perovskite film prior to fluoropolymer deposition and the bottom line represents the perovskite film after the deposition of the fluoropolymer from scCO_2_/ethanol. Comparison between the two spectra shows clear differences. The bands observed near 800 cm^−1^ and 950 cm^−1^ in the bare perovskite are associated with the intramolecular modes of methylammonium (MA) [30]. These bands decrease significantly in the sample annealed in scCO_2_/ethanol with the fluoropolymer solute. One possible explanation for the reduction of these bands is preferential extraction of methylammonium, which is soluble in ethanol. Another significant difference in the two spectra is the appearance of a strong peak at 1100 cm^−1^ in the Raman spectrum for the sample annealed in scCO_2_/ethanol with the fluoropolymer solute. This peak is not present in the uncoated perovskite sample and is associated with the C-F stretching mode of the poly(TFEMA) polymer [31].

**Figure 4:**
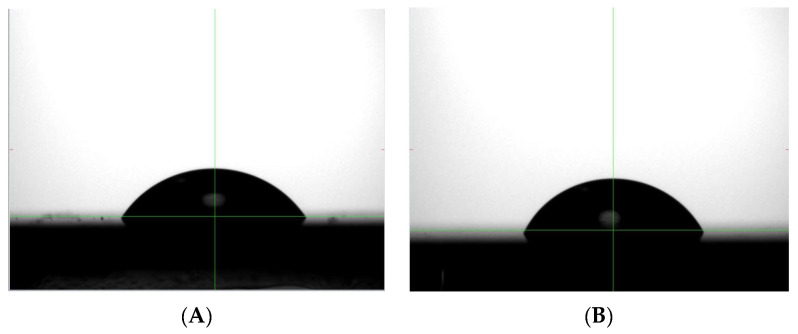
Contact angle measurements on (**A**) perovskite and (**B**) poly(TFEMA) film on perovskite.

One additional indirect test for the presence of fluoropolymer on the perovskite surface is an increase in hydrophobicity. Poly(TFEMA) is hydrophobic and the presence of even a small amount of the fluoropolymer on the perovskite surface would be expected to change the surface wettability. Figure 4A shows the profile of a water droplet placed onto the surface of the bare perovskite film and Figure 4B shows the profile of a water droplet placed onto the surface of the perovskite after exposure to the scCO_2_/ethanol solution containing poly(TFEMA). The contact angle increased from 56° for the uncoated perovskite sample to 62° for the sample after fluoropolymer deposition. While not conclusive, this small increase in surface hydrophobicity is an indirect indication of the presence of poly(TFEMA).

The ability to incorporate chemical additives into perovskite films for surface protection or defect passivation could potentially be used to increase the long-term performance of solar cells based on these materials. The purpose of this study was to demonstrate that supercritical fluids have the potential to be used to incorporate fluoropolymers or other molecules into perovskite films during crystallization. The surface characterization performed in this initial investigation (SEM, Raman, and contact angle) all appear to indicate the presence of fluoropolymer. However, the fluoropolymer does not appear as distinct particles, but the evidence appears to show that the fluoropolymer is incorporated or integrated within the perovskite film during annealing in a supercritical fluid. These early results are therefore promising, but much additional work is needed in order to produce high-quality surfaces compatible with use in perovskite solar cells followed by photovoltaic and stability testing to see if the fluoropolymer imparts any benefit in terms of performance longevity.

## 3. Methods

### 3.1. Substrate Preparation

CH_3_NH_3_PbI_3_ perovskite coatings were deposited on 25 mm by 25 mm fluorine-doped tin oxide (FTO) glass substrates (Ossila, TEC 15). Substrate cleaning included washing with detergent and deionized water, ultrasonic bath in 2% Hellmanex solution, rinsing with deionized water, ultrasonic bath in isopropanol for 15 min, ultrasonic bath in acetone for 15 min, rinsing with acetone, rinsing with isopropanol, drying the isopropanol with dry air, and plasma cleaning for 4 min.

### 3.2. Perovskite Layer

The perovskite (CH_3_NH_3_PbI_3_) film was synthesized from a 1:1:1 molar ratio of 2.385 g methyl ammonium iodide (CH_3_NH_3_I) (98%, Sigma-Aldrich, St. Louis, MO, USA), 6.915 g lead (II) iodide (PbI_2_) (99.9985%, Alfa Aesar, Haverhill, MA, USA); and 1.063 mL dimethyl sulfoxide (DMSO) (>99.9%, anhydrous, Sigma-Aldrich), 9.484 mL N,N-dimethylformamide (DMF) (>99.8%, anhydrous, Sigma-Aldrich), and 0.3 mL diethyl ether (>99.8%, anhydrous, Sigma-Aldrich). The solution was stirred for one hour at room temperature and filtered using a 0.2 μm syringe filter (Corning Inc., Corning, NY, USA). The solution was then spin-coated onto substrate at 6000 rpm for 25 s and 0.6 mL diethyl ether was dripped onto the rotating surface 6 sec into spinning. Spin coating was carried out in an argon-filled glove box. Substrates were then placed on a hot plate at 50 °C for 30 min.

### 3.3. Cloud Point Determination

**Figure 5:**
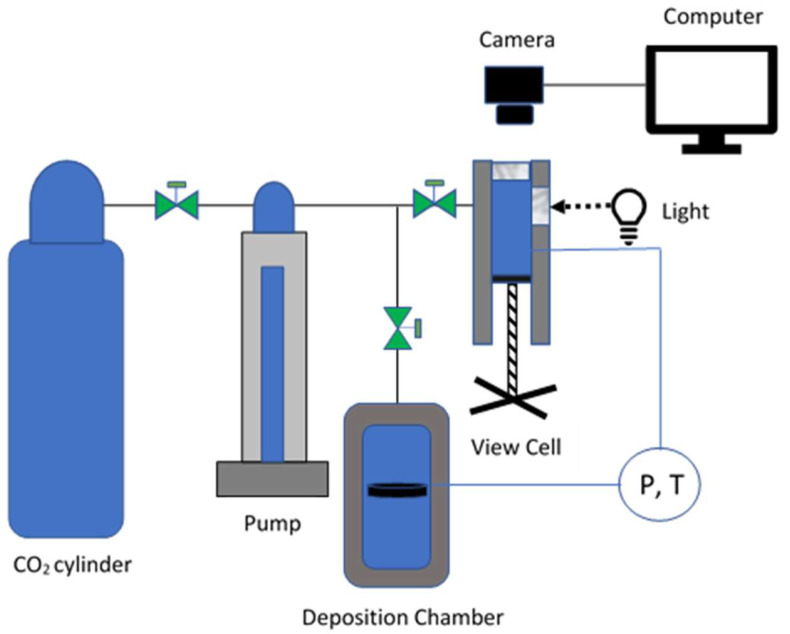
Schematic diagram illustrating the primary components of the phase behavior and deposition chamber apparatus.

Figure 5 is a schematic diagram illustrating the primary components of the high-pressure system used in this study. The system includes a variable volume view cell for cloud point determination and a separate chamber for perovskite annealing and fluoropolymer deposition. Cloud point measurements are widely used for the determination of polymer solubility in supercritical fluids [32]. The cloud point was determined for the mixture of 0.02 weight percent ethanol, 0.03 weight percent Poly(TFEMA) and 0.95 weight percent of scCO_2_ utilizing a Supercritical Fluid Technologies, Inc., Newark, DE, USA (SFT) Phase Monitor. We loaded 3.54 g of poly(TFEMA) and 3 mL of ethanol into the SFT-Phase monitor 30 cc processing vessel. The light control dial was adjusted until the sample could be viewed through a quartz window, and images were captured using a CCD camera through a quartz window connected to a monitor.

**Figure 6:**
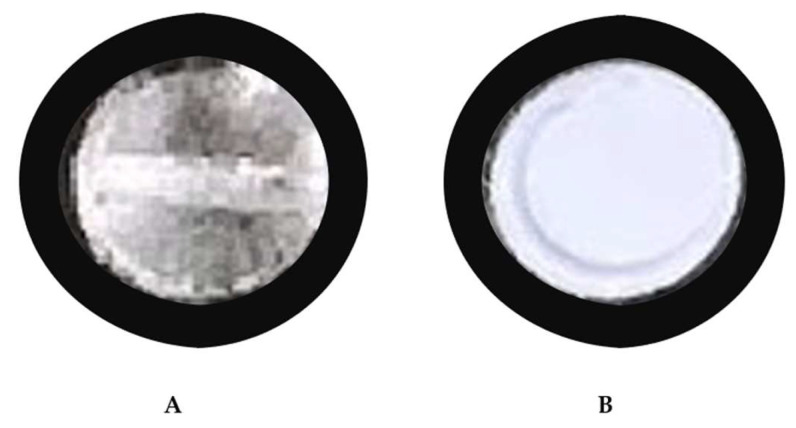
Camera images showing (**A**) dissolved (stir bar seen) and (**B**) precipitating polymer (cloudy) for cloud point determination.

The cell was slowly filled with carbon dioxide with a helium headspace and siphon tank (Airgas), then heated to 31 °C using flexible heating cords wrapped around the cylinder. The pressure and temperature of the vessel were measured to determine the CO_2_ density, and the mass was determined to within 2% accuracy from the known volume of the vessel. Once the temperature reached 31 °C, the cell volume was isothermally decreased until the pressure reached 1500 psi. Figure 6A is an image of the clear solution indicating that the fluoropolymer was completely dissolved. The volume of the cell was then slowly and isothermally increased causing a corresponding decrease in pressure. The solution became completely cloudy, as shown in Figure 6B, at a pressure of 900 psi as the polymer precipitated from solution.

### 3.4. Polymer Deposition

The perovskite films fabricated as described in Section 3.2 were placed into a 600 mL pressure vessel (Parr instrument Pressure Reactor 4768). The polymer and solvent concentrations used during the cloud point measurements were used for film deposition: 0.02 weight percent ethanol, 0.03 weight percent Poly(TFEMA) and 0.95 weight percent of scCO_2_. 7.086 g of poly(TFEMA) and 6 mL of ethanol were measured and loaded into the bottom of the pressure vessel and a syringe pump (Teledyne ISCO Pump 260D) was used to pressurize the chamber with CO_2_ to dissolve the ethanol and poly(TFEMA). Resistive heating straps wrapped around the exterior of the pressure vessel were used to control the temperature and the experiment was carried out at a temperature of (31 °C) and pressure of 1000 psi as informed by the cloud point measurements of Section 3.3. The pressure and temperature were held for 10 min and the pressure was then slowly decreased over 10 min. The sample was then removed and characterized.

## 4. Conclusions

A new process is reported for the incorporation of a fluoropolymer additive into a perovskite thin film. The fluoropolymer was incorporated into methylammonium lead iodide perovskite films by annealing the films in scCO_2_/ethanol solvent with a poly(TFEMA) solute. The film morphology, chemistry, and wettability were characterized before and after fluoropolymer exposure and suggest that the polymer was incorporated into the film rather than deposited in the form of a particulate layer on the surface. The incorporation of a fluoropolymer into a perovskite film has the potential to enhance the moisture resistance of perovskite-based solar cells.

## Data Availability

The data presented in this study are available on request from the corresponding author.

## Sample Availability

Samples of the compounds “perovskite films” are available from the authors.
